# Counterfactual Explanation of Brain Activity Classifiers Using Image-To-Image Transfer by Generative Adversarial Network

**DOI:** 10.3389/fninf.2021.802938

**Published:** 2022-03-16

**Authors:** Teppei Matsui, Masato Taki, Trung Quang Pham, Junichi Chikazoe, Koji Jimura

**Affiliations:** ^1^Department of Biology, Okayama University, Okayama, Japan; ^2^JST-PRESTO, Japan Science and Technology Agency, Tokyo, Japan; ^3^Graduate School of Artificial Intelligence and Science, Rikkyo University, Tokyo, Japan; ^4^Supportive Center for Brain Research, National Institute for Physiological Sciences, Okazaki, Japan; ^5^Araya Inc., Tokyo, Japan; ^6^Department of Biosciences and Informatics, Keio University, Yokohama, Japan

**Keywords:** fMRI, deep learning, explainable AI, decoding, generative neural network, counterfactual explanation

## Abstract

Deep neural networks (DNNs) can accurately decode task-related information from brain activations. However, because of the non-linearity of DNNs, it is generally difficult to explain how and why they assign certain behavioral tasks to given brain activations, either correctly or incorrectly. One of the promising approaches for explaining such a black-box system is counterfactual explanation. In this framework, the behavior of a black-box system is explained by comparing real data and realistic synthetic data that are specifically generated such that the black-box system outputs an unreal outcome. The explanation of the system's decision can be explained by directly comparing the real and synthetic data. Recently, by taking advantage of advances in DNN-based image-to-image translation, several studies successfully applied counterfactual explanation to image domains. In principle, the same approach could be used in functional magnetic resonance imaging (fMRI) data. Because fMRI datasets often contain multiple classes (e.g., multiple behavioral tasks), the image-to-image transformation applicable to counterfactual explanation needs to learn mapping among multiple classes simultaneously. Recently, a new generative neural network (StarGAN) that enables image-to-image transformation among multiple classes has been developed. By adapting StarGAN with some modifications, here, we introduce a novel generative DNN (counterfactual activation generator, CAG) that can provide counterfactual explanations for DNN-based classifiers of brain activations. Importantly, CAG can simultaneously handle image transformation among all the seven classes in a publicly available fMRI dataset. Thus, CAG could provide a counterfactual explanation of DNN-based multiclass classifiers of brain activations. Furthermore, iterative applications of CAG were able to enhance and extract subtle spatial brain activity patterns that affected the classifier's decisions. Together, these results demonstrate that the counterfactual explanation based on image-to-image transformation would be a promising approach to understand and extend the current application of DNNs in fMRI analyses.

## Introduction

Recent studies demonstrated promising results of the deep neural network (DNN) (LeCun et al., 2015) for decoding cognitive or behavioral information from brain activity images as observed with functional magnetic resonance imaging (fMRI) (Wang et al., 2020; Tsumura et al., 2021). However, despite these promising results, further applications of DNN to fMRI data could be limited due to its poor interpretability. Because of its highly non-linear and complex processing, it is often difficult to interpret what features of a given input led to the DNN's decision (Dong et al., 2019). For example, in the case of brain activity decoding, even though the DNN can accurately assign brain activations to a particular task, it is difficult to pinpoint which patterns of brain activations were important for the DNN's decisions. Such interpretability would be even more important when the DNN's decoding is incorrect. Gradient-based visualization methods, such as Grad-CAM (Selvaraju et al., 2020), are frequently used to highlight image regions potentially relevant for the DNN's decision [see Tsumura et al. (2021) for an application in neuroimaging]. However, several limitations of the gradient-based methods, such as high numbers of false positives (Eitel and Ritter, 2019), have been reported. Thus, alongside improving the gradient-based methods (Chattopadhay et al., 2018), it would be beneficial to explore alternative approaches for interpreting the inner workings of DNNs (Adadi and Berrada, 2018).

**Figure 1:**
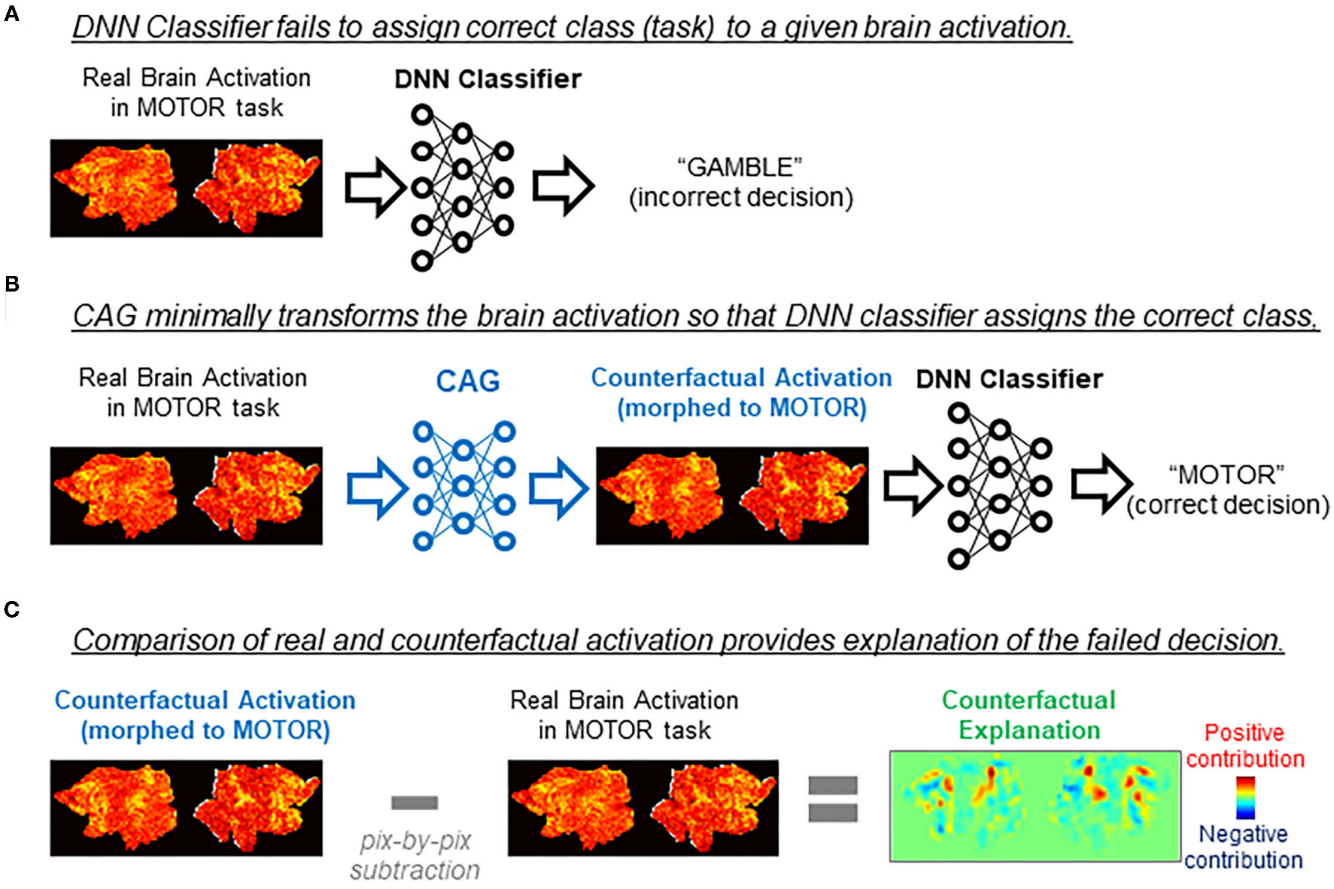
Applications of counterfactual explanation in fMRI. The example illustrates an application of counterfactual explanation to a misclassification by a DNN classifier. **(A)** In this example, a DNN classifier incorrectly assigned EMOTION to a map of brain activation obtained in a MOTOR task. Because of the black-box nature of the DNN classifier, it is difficult to explain why the misclassification occurred. **(B)** A generative neural network for counterfactual brain activation (CAG) minimally transforms the real brain activation in **(A)** so that the DNN classifier now assigns MOTOR to the morphed activation (counterfactual activation). **(C)** Counterfactual explanation of misclassification in **(A)** can be obtained by taking the difference between the real activation and the counterfactual activation. In this example, the real brain activation would have been classified (correctly) as MOTOR if red (blue) brain regions in the counterfactual explanation had been more (less) active.

Counterfactual explanation is one of the major approaches for explaining DNN's inner working (Goyal et al., 2019; Wang and Vasconcelos, 2020). To explain how the decision on a given data was made, counterfactual explanation uses artificial data (“counterfactuals”) that are generated from the real data but targeted to an unreal outcome (decision). By comparing the DNN's decision on the real data and the counterfactual, one can deduce explanations of the decisions made by the DNN. For example, we consider a case in which a brain activity classifier incorrectly assigns a gambling task to a brain activation produced in a motor task (Figure 1A). We consider a minimal transformation of the original brain activation to a counterfactual activation that is classified (correctly) as a motor task activation by the DNN classifier (Figure 1B). By directly comparing the original brain activation and the counterfactual activation, one can explain the classifier's decision by making a statement such as “This brain activation map would have been correctly classified to the gambling task if brain areas X and Y had been activated.” (Figure 1C). As in this example, counterfactual explanation can provide intuitive explanations of a black-box decision system without opening the black-box, which is a critical aspect of the technique.

Although the generation of counterfactuals for high-dimensional data such as natural images and medical images had been difficult, recent advancement in DNN-based image generation has made counterfactual explanation applicable to these domains. For natural images, several studies have successfully used counterfactual explanation to explain the behavior of DNN-based image classifiers (Chang et al., 2019; Liu et al., 2019; Singla et al., 2020; Zhao, 2020). In medical image analyses, counterfactual explanation has also been applied to DNN-based classifiers of X-ray and structural MR images (Mertes et al., 2020; Pawlowski et al., 2020). However, to the best of our knowledge, counterfactual explanation has not been utilized for DNN-based classifiers of fMRI data. The lack of application to fMRI data may be due to the fact that commonly used fMRI dataset [such as data distributed by the Human Connectome Project (Van Essen et al., 2013)] usually contains data for multiple tasks. Because of this characteristic of fMRI dataset, unlike commonly used image generator that performs image-to-image transformation only between two classes, a generator of counterfactual brain activations needs to be able to transform the inputs to more than three classes.

Recently, the StarGAN (Choi et al., 2018) has enabled image-to-image transfer among multiple classes, thus opening the possibility to extend the application of counterfactual explanation to the multiclass fMRI dataset. In this study, based on the StarGAN model, we developed a generative neural network named counterfactual activation generator (CAG), which provides counterfactual explanations for a DNN-based classifier of brain activations observed with fMRI. This study aims to provide a proof of principle that counterfactual explanation can be applied to fMRI data and the DNN-based classifiers of brain activations. We demonstrated several applications of CAG. First, CAG could provide counterfactual explanations of correct classifications of the brain activations by DNN-based classifiers. Specifically, the counterfactual explanation highlighted the patterns of brain activations that were critical for the DNN classifier to assign the brain activations to particular tasks. Similarly, CAG could provide counterfactual explanations of incorrect classifications by DNN-based classifiers. Moreover, iterative application of CAG accentuated and extracted subtle image patterns in brain activations that could strongly affect the classifier's decisions. These results suggest that the image transfer-based methods, such as CAG, would be a powerful approach for interpreting and extending DNN-based fMRI analyses.

## Materials and Methods

### Datasets

**Figure 2:**
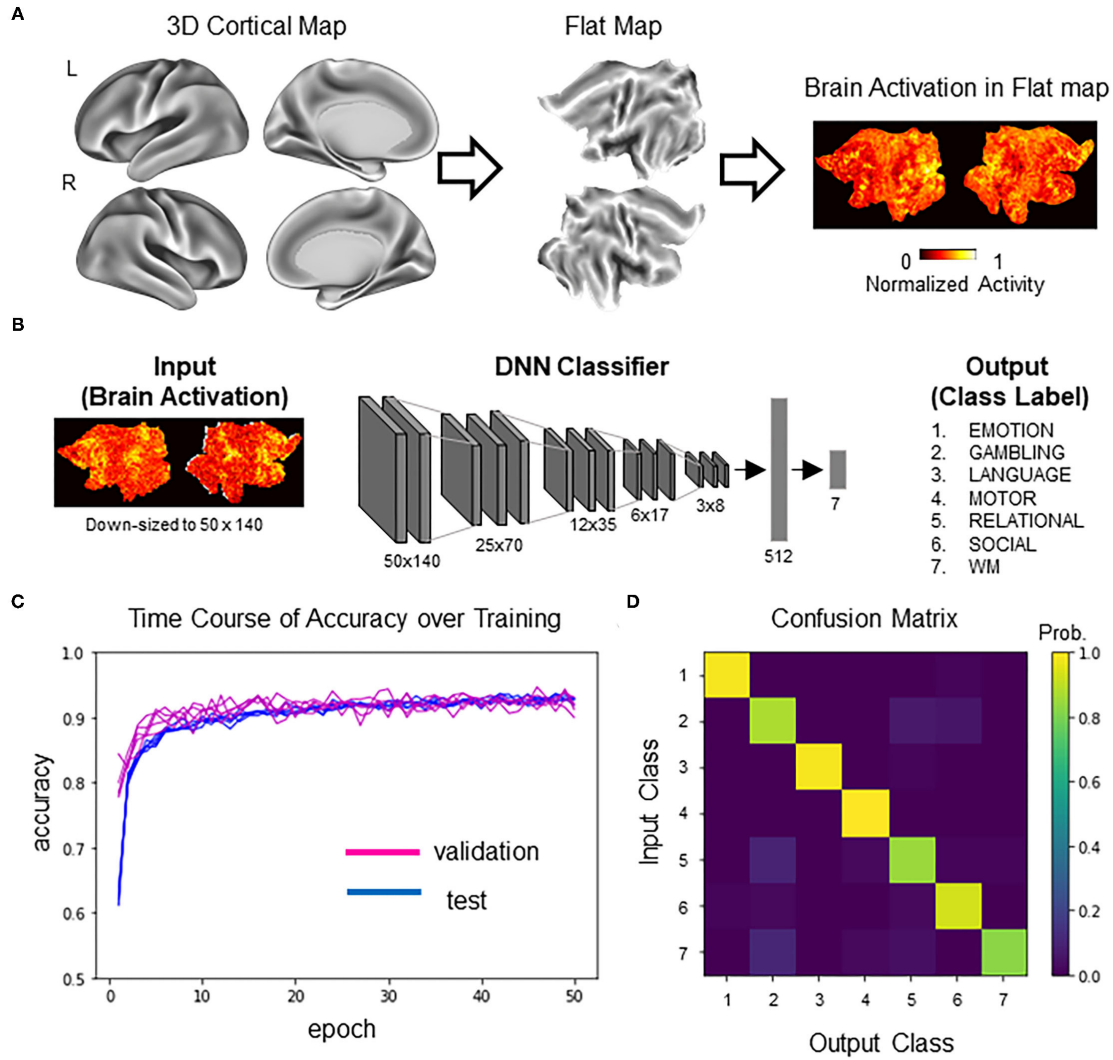
DNN classifier for brain activity decoding. **(A)** Following the standard procedure developed by HCP (Glasser et al., 2016), neocortex in the two hemispheres was mapped to two cortical sheets. Each neocortical activity image was mapped to the two sheets, which was then input to the DNN classifier (for details, see Tsumura et al., 2021). **(B)** Model architecture of the DNN classifier. The input was a picture containing two sheets of cortical activations. The picture was downsampled for later processing by the generative neural network. The DNN classifier was a deep convolutional network similar to the one described in our previous study (Tsumura et al., 2021). The output of the DNN classifier was one-hot vectors representing seven behavioral tasks in the HCP dataset. **(C)** Training history of the transfer learning. Test accuracy (blue) and validation accuracy (magenta) are shown for five replicates. Note that the chance level is 14.3% (1/7). **(D)** Profile of the classifier's decision (confusion matrix) in the validation set.

Training data were single-subject second-level z-maps obtained during the performance of seven behavioral tasks from the S1200 release of the Human Connectome Project (*N* = 992; HCP; http://www.humanconnectomeproject.org/) (Barch et al., 2013; Van Essen et al., 2013; Glasser et al., 2016). From each participant, statistical z-maps were obtained for activation contrasts for the emotional processing task (face vs. shape), the gambling task (reward vs. loss), the language processing task (story vs. math), the motor task (average of all motions), the relational processing task (relational processing vs. matching), the social cognition task (mental vs. random), and the N-back working memory task (2-back vs. 0-back). For brevity, the seven tasks are denoted as follows: (1) EMOTION, (2) GAMBLING, (3) LANGUAGE, (4) MOTOR, (5) RELATIONAL, (6) SOCIAL, and (7) WORKING MEMORY (WM). We used gray-scaled flat 2D cortical maps (Glasser et al., 2016) provided from HCP for dimensional compatibility of images between VGG16-ImageNet and activation maps. The flattened maps were created using the Connectome Workbench (https://www.humanconnectome.org/software/connectome-workbench/) following a procedure described in (Tsumura et al., 2021) (Figure 2A).

### DNN Classifier of Brain Activations

The DNN classifier of brain activations used in this study was adapted from our previous study (Tsumura et al., 2021) (Figure 2B). Briefly, the DNN classifier was based on VGG16 (Simonyan and Zisserman, 2015), with five convolution layers for extracting image features and two fully connected layers for classification of the seven tasks. Initial parameters of convolution layers were set to parameters pretrained with concrete object images provided from ImageNet (Simonyan and Zisserman, 2015) (http://www.image-net.org/). The VGG16/ImageNet model is capable of classifying concrete object images into 1,000 item categories. Importantly, it has been demonstrated that the pretrained model can learn novel image sets more efficiently than the non-trained model by tuning convolution and fully connected layers and fully connected layers only (Pan and Yang, 2010). Thus, the current analysis retrained the pretrained VGG16-ImageNet model to classify brain activation maps. To enable processing by generative neural networks described below, activation maps were spatially downsampled from 570 by 1,320 pixels to 50 by 140 pixels. Data were split into training data (*N* = 4,730) and validation data (*N* = 518) (note that some participants in the dataset did not complete all seven tasks). Training was conducted using the training data with ten-fold crossvalidation. Hyperparameters for the training were as follows: batch size, 10; epoch, 50; learning rate, 0.0001; optimizer, stochastic gradient descent (SGD); loss function, categorical crossentropy. Pixels outside of the brain were set to zero. Model training and testing were implemented using Keras (https://keras.io/) under a Tensorflow backend (https://www.tensorflow.org/). Five instances of the DNN classifier were trained for replication. All parameters, including the training data, were the same for all the replicates.

### Counterfactual Activation Generator (CAG)

**Figure 3:**
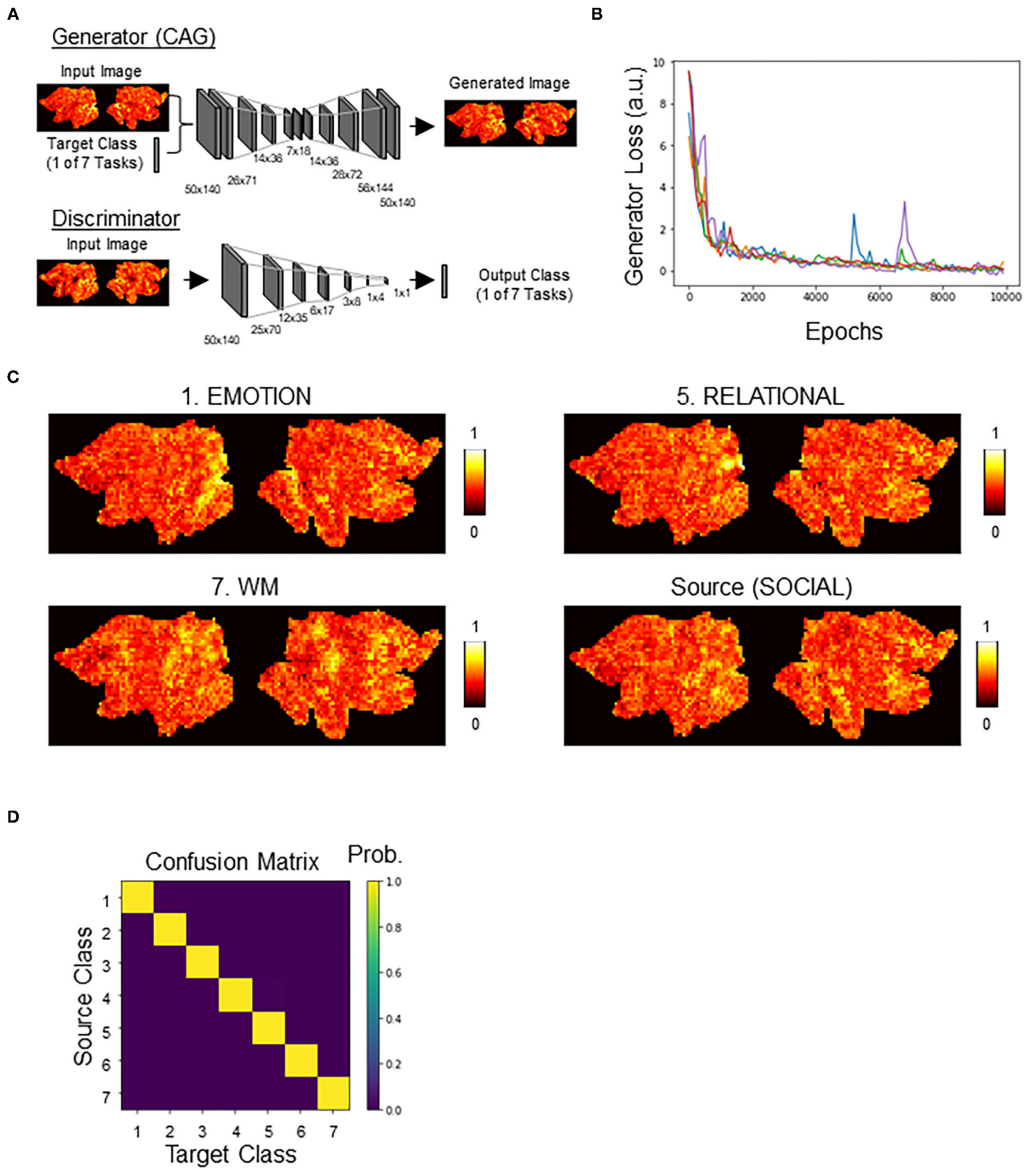
Counterfactual activation generator (CAG). **(A)** Generator and discriminator architectures, see Methods for details. Networks were modified from StarGAN. Generator, once trained, served as CAG. Generator takes a combination of an image of brain activation and a one-hot label indicating the target class as an input. Generator outputs a counterfactual brain activation that is a minimal transform of the input brain activation toward the target class. Discriminator takes an activation map output by generator and outputs a one-hot label. Discriminator was cotrained with generator, as in StarGAN. **(B)** Time courses of the generator loss. Different colors indicate different replicates (*n* = 5). **(C)** Representative counterfactual activations generated by CAG. All counterfactual activations were generated from the source activation. See Supplementary Figure 3 for transformation to all categories. **(D)** Confusion matrix showing the classifier's decision profile on counterfactual activations.

We adopted the architecture of StarGAN (Choi et al., 2018), consisting of discriminator and generator, with a modification to add a new loss term for the DNN classifier (Figure 3A; see also Supplementary Figure 1 for an illustration of our overall approach). Except for this addition of the new loss term (CAG loss), other parameters were the same as in the original StarGAN model. Briefly, the goal was to train a single generator that learns mapping among multiple classes (in this case, the seven HCP tasks). We regarded this generator as CAG. To achieve this, we trained CAG to transform a brain activation ***x*** with class-label *y* (source class) to a perturbation toward *y*^*c*^ (target class), such that *CAG*(*x*, *y*^*c*^) → *x*^*c*^. An auxiliary discriminator was introduced to allow a single discriminator to control multiple classes. Thus, the discriminator produced the probability distributions over both the source and the target classes, *D*:*x* → {*D*_*src*_(*x*), *D*_*cls*_(*x*)}.

The loss terms were described as follows. Wasserstein loss $\left(L_{wass}\right)$ and gradient penalty loss $\left(L_{gp}\right)$ were included to make the generated brain activations indistinguishable from the real brain activations. $L_{wass}$ between the real and counterfactual activations and $L_{gp}$ were defined as follows:


$$\begin{array}{l} L_{wass}\;=\;E_{x}\left[D_{src}\left(x\right)\right]-\;E_{x,c}\left[D_{src}\left(CAG\left(x,c\right)\right)\right]\\ L_{gp}\;=\;E_{\hat{x}}\left[{\left(∥\nabla_{\hat{x}}\;D_{src}\left(\hat{x}\right){∥}_{2}-1\right)}^{2}\right] \end{array}$$

where $\hat{x}$ was sampled uniformly along a straight line between a pair of a real and a generated activation.

Domain classification loss was included to ensure that the transformed activation was properly classified as the target class. We considered two types of objectives. The first one is a domain classification loss of real activations used to optimize discriminator $\left(L_{cls}^{r}=\;E_{x,c^{{}^{\prime }}}\left[-\log\;D_{src}\left(c^{\prime }|x\right)\right]\right)$. The second one is a domain classification loss of fake activations used to optimize CAG $\left(L_{cls}^{f}=\;E_{x,c}\left[-\log\;D_{src}\left(c|CAG\left(x,c\right)\right)\right]\right)$. This loss term forced CAG to generate activations that could be classified as the target classes.

Reconstruction loss $\left(L_{rec}\right)$ was defined using the cycle consistency loss (Kim et al., 2017; Zhu et al., 2017) as follows,


$$\begin{array}{l} L_{rec}\;=\;E_{x,c,c^{{}^{\prime }}}\left[\left(∥x-\;\text{CAG}\left(\text{CAG}\left(x,\text{c}\right),{\text{c}}^{\prime }\;\right){∥}_{1}\right)\right] \end{array}$$

where CAG tried to reconstruct the original activation from the transformed activation.

Additionally, we included a loss term for the DNN classifier $\left(L_{cnn}\right)$ to force the mappings learned by CAG to be aligned with the classifier's decisions. This loss term was calculated using categorical crossentropy over the fake activations.

The total losses for discriminator $\left(L_{D}\right)$ and the CAG loss $\left(L_{G}\right)$ were defined using the loss terms as follows:


$$\begin{array}{l} L_{D}=\;L_{wass}^{r}+\;L_{wass}^{f}+\;\lambda_{gp}L_{gp}+\;\lambda_{cls}L_{cls}^{r}\\ L_{G}=\;L_{wass}+\;\lambda_{cls}L_{cls}^{f}+\;\lambda_{rec}L_{rec}+\;\lambda_{cnn}L_{cnn} \end{array}$$

where $L_{wass}^{r}$ and $L_{wass}^{f}$ stand for Wasserstein loss for real and fake activations, respectively. We used the same hyperparameters and procedures used in the original StarGAN model, except for λ_*cnn*_ which was newly introduced in CAG. Instance normalization was used for the generator, but no normalization was used for the discriminator. The generator network consisted of three convolutional layers for downsampling, followed by two convolutional layers (replacing two residual blocks in the original StarGAN model), which was intern followed by four convolutional layers for up sampling. We used λ_*gp*_ = 10, λ_*cls*_ = 1, λ_*rec*_ = 10, and λ_*cnn*_ = 1 for all experiments. All models were trained using Adam (Kingma and Ba, 2014), with β_1_ = 0.5 and β_2_ = 0.999. Training was done using the training data with ten-fold crossvalidation for 10,000 epochs. Batch size and learning rate were set to 16 and 0.0001, respectively, in all experiments. The code for CAG is available upon reasonable request to the corresponding author.

### Counterfactual Explanation of Correctly and Incorrectly Classified Images

Counterfactual explanation of correctly classified images was performed on the correctly classified brain activations (*N* = 478 out of 518 that were not used in the classifier training). Each counterfactual explanation was set to explain “Why this activation was correctly classified as class (task) A instead of class B?” To do this, the original brain activation was transformed by CAG toward class B. Counterfactual explanation was obtained by pixel-by-pixel subtraction of the original activation from the counterfactual activation. As for counterfactual explanation of correct classifications, counterfactual activations were obtained by transforming the correctly classified activations to one of the randomly chosen incorrect classes.

To quantitatively evaluate the effectiveness of counterfactual explanations, we conducted two analyses. In the first analysis, we perturbed image transformation by CAG at various levels and examined its effect on the classifier's decisions. For the perturbation, pixels in each counterfactual explanation whose values were below a chosen percentile threshold (α) were set to zero (*CE*_α_). Then, the perturbed counterfactual explanation was added back to the original activation (*Activation*_*original*_) as follows:


$$\begin{array}{l} Activation_{new}=Activation_{original}+CE_{\;\alpha \;} \end{array}$$

The resulting activation (*Activation*_*new*_) was normalized to have minimum and maximum values of zero and one, respectively, and then input to the DNN classifier. The percentile threshold (α) took values ranging from 0 to 100% with a 20% step. Note that *Activation*_*new*_ is equal to the counterfactual activation and *Activation*_*original*_ when α equals to 0 and 100%, respectively. In the second analysis, each counterfactual explanation was compared with a “control explanation,” which was calculated as the difference between the true class's average activations and the target class used for the transformation (Δ* Ave*). The control explanation was added to the original activation (*Activation*_*original*_) as follows,


$$\begin{array}{l} Activation_{new}=Activation_{original}+\;\Delta \;Ave\times \;\kappa \end{array}$$

The resulting activation (*Activation*_*new*_) was normalized to have minimum and maximum values of zero and one, respectively, and then input to the DNN classifier. The parameter for mixing (κ) took values ranging from 0 to 5 at with a 0.1 step and was adjusted individually for each control explanation to maximize the total number of cases classified to the target class used for transformation.

Counterfactual explanation of incorrectly classified images was performed similarly on each incorrectly classified brain activation (*N* = 40). Each counterfactual explanation was set to explain “Why this activation was incorrectly classified as class (task) B instead of class A?” To do this, the original brain activation was transformed by CAG toward the true class A. Counterfactual explanation was obtained by pixel-by-pixel subtraction of the original activation from the counterfactual activation. The two quantitative analyses for the counterfactual explanation of correct classifications were similarly applied to the counterfactual explanation of incorrect classifications. In these analyses, the target class for the image transformation by CAG was set to the correct classes (instead of randomly chosen classes in the case of correct classification).

### Counterfactual Exaggeration and Feature Extraction

Counterfactual exaggeration (Singla et al., 2020) was performed by iteratively transforming a real brain activation toward one class. We performed up to eight iterations. Feature extraction was done by subtracting the third iteration from the eighth iteration. To quantitatively evaluate the extracted feature, the feature was added to each activation in the validation set (*N* = 518), and then, the summed image was input to the DNN classifier. For 12 extracted features from randomly chosen activations, the percent of activations assigned to the added feature's class were calculated.

## Results

### DNN Classifier Decoded Task Information From Brain Activity With High Accuracy

We first trained a DNN classifier that was used as the target for counterfactual explanation. Brain activations were converted to flattened maps, which were then input to the DNN classifier (Figure 2A). The DNN classifier was based on VGG16 pretrained on the ImageNet dataset (Tsumura et al., 2021) (Figure 2B). The pretrained DNN classifier was trained to classify brain activation maps using transfer learning (Pan and Yang, 2010). After 50 epochs of training, the DNN classifier reached ~92% of classification accuracy for the held-out validation data. Similar results were obtained for a total of five replicates, suggesting high reproducibility (Figure 2C). Figure 2D shows the confusion matrix showing the classifier's decision profile (see also Supplementary Table 1 for exact values). Similar confusion matrices were obtained for all the replicates (data not shown). These results suggest that DNN classifiers could accurately decode task information from individual brain activations.

### CAG Generated Counterfactual Activations Were Realistic and Fooled the Classifiers

We next trained a generative neural network (CAG) for counterfactual explanations of the DNN classifier's decisions. For this, we adopted, with modifications, the architecture of StarGAN (Choi et al., 2018) that can perform image-to-image transformation among multiple classes. Two DNNs, generator (CAG) and discriminator, were simultaneously trained (Figure 3A; Supplementary Figure 1). By including the classification loss by the DNN classifier, CAG was trained to simultaneously fool both the discriminator and the DNN classifier (Supplementary Figure 1; see Methods for details). Throughout the training, the generator loss, which is a good indicator of the quality of the generated image (Arjovsky et al., 2017), consistently decreased toward zero and plateaued around 10,000 epochs of training (data for five replicates are shown in Figure 3B; see also Supplementary Figure 2 for time courses of all the loss terms). After the training, CAG could transform a real brain activation into a counterfactual brain activation that was visually indistinguishable from the real activations (Figure 3C; Supplementary Figure 3). The training of the CAG was also designed such that the CAG transformed an input activation map to any of the seven classes and fool the DNN classifier. Thus, the trained DNN assigned the targeted class to the counterfactual activations at almost 100% accuracy (Figure 3D; Table 1). These results suggest that CAG fulfilled the goal of generating counterfactual brain activations that were not only visually realistic but also fooled the DNN classifier.

**Table 1:** Decision profile of DNN classifier on counterfactual activations.

**CLASS**	**EMOTION**	**GAMBLING**	**LANGUAGE**	**MOTOR**	**RELATIONAL**	**SOCIAL**	**WM**
N_correct_ (%)	518 (100%)	518 (100%)	518 (100%)	512 (98.8%)	518 (100%)	518 (100%)	518 (100%)

*Each image in the validation set (N = 518) was morphed toward one of the seven classes and then input to the DNN classifier*.

### Counterfactual Explanation of Misclassification by DNN Classifiers

**Figure 4:**
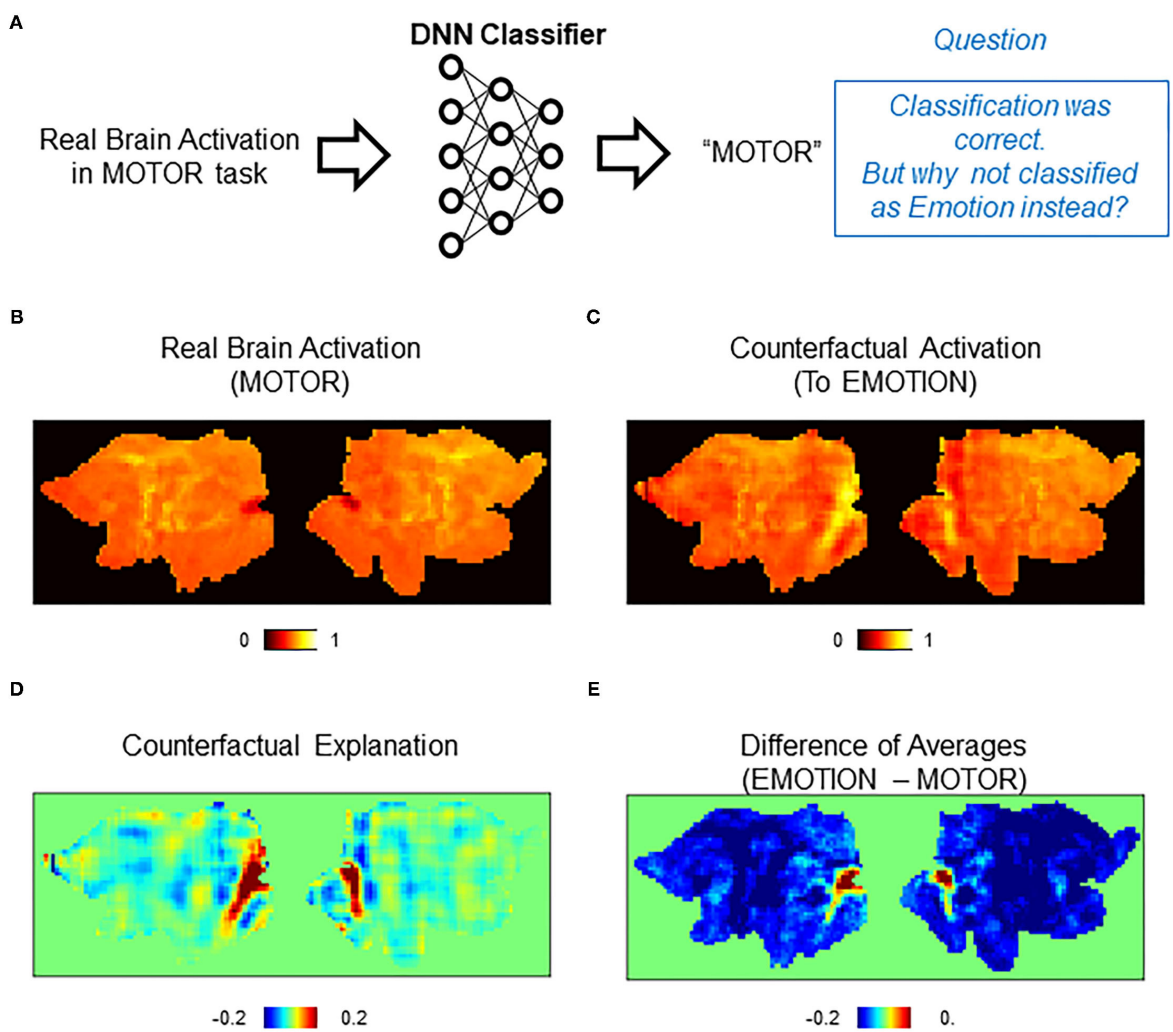
Counterfactual explanation of correct classification. **(A)** Schematics of the question asked in this analysis. In this example, the DNN classifier correctly assigned a “MOTOR” label to a real brain activation in the MOTOR task. Here, we want to interrogate this correct decision. Specifically, we ask a question “why did the classifier assign MOTOR instead of EMOTION?” **(B–E)** Examples of counterfactual explanation. **(B)** A population average map of real brain activation in the MOTOR task. **(C)** A population average map of counterfactual activation obtained by transforming the map in **(A)** to EMOTION. Transformation was conducted for each activation map and then averaged across the population. **(D)** Pixel-by-pixel subtraction of maps in **(B)** and **(A)** that serves as counterfactual explanation. This map explains why the map was classified as MOTOR but not EMOTION. **(E)** Simple difference between the average of real activations in the EMOTION and MOTOR tasks.

Using CAG, we first conducted counterfactual explanation of the classifier's correct decisions. Specifically, we tried to visualize the pattern of brain activation that led the classifier to assign the correct class but not another (incorrect) class (Figure 4A). In the first example, brain activations correctly classified as MOTOR by the DNN classifier were examined (Figure 4B). We asked why these activations were not classified as EMOTION. To see this, a counterfactual activation was created by transforming each original activation toward EMOTION using CAG (Figure 4C). Then, the counterfactual explanation was obtained by taking the difference between the original and the counterfactual activations (Figure 4D). The positive and negative regions in the counterfactual explanation were the regions that had positive and negative influence, respectively, on the classifier's decision of assigning EMOTION but not MOTION to the counterfactual activation. In other words, the DNN classifier would have classified the original activation as EMOTION if the positive regions in the counterfactual explanation had been more active (and the opposite for the negative regions).

To quantitatively evaluate the counterfactual explanation, we compared it against a difference between the population-averaged activations of EMOTION and MOTOR (average of 74 and 75 activations, respectively) (Figure 4E). Whereas the difference of average maps highlighted a small portion of brain areas in the occipital cortex, counterfactual explanation additionally found lateral temporal areas to be relevant. Because lateral temporal areas are known to be activated by emotional and facial processing (White et al., 2014; Glasser et al., 2016), it is reasonable to find these areas highlighted in the counterfactual explanation. The reason that only the occipital cortex was highlighted in the difference in average maps is most likely due to very high activation in this area in the average map of EMOTION compared to the average map of MOTOR. These differences between the counterfactual explanation and the explanation by the difference of averages can be understood as the difference between univariate and multivariate analyses (Jimura and Poldrack, 2012). The average map that is derived from the univariate analysis (pixel-based GLM) is affected by the choice of particular control conditions, and thus, the explanation by the difference of the averages would be affected by difference in the control conditions. In contrast, the counterfactual explanation can robustly detect relevant activations using spatial patterns of multiple pixels. Consistent with this idea, the counterfactual explanation, but not the difference of average maps, successfully highlighted the orbitofrontal areas implicated for emotional processing (Figure 4D) (Goodkind et al., 2012; Rolls et al., 2020).

The second example shows counterfactual explanation of why WM activations were not classified as LANGUAGE (Supplementary Figure 4). In this case, unlike the previous example, the difference of averages of WM and LANGUAGE highlighted large portions of the brain (red regions in Supplementary Figure 4; average of 75 and 73 maps, respectively, for WM and LANGUAGE). With such large and distributed areas being highlighted, it is difficult to pinpoint particular areas without arbitrary thresholding. In contrast, the counterfactual explanation highlighted distributed but much more localized brain areas (Supplementary Figure 4C). It is evident from the counterfactual explanation that activations in frontal and temporal brain areas would have been necessary to shift the DNN classifier's decision from WM to LANGUAGE.

Out of 478 correctly classified validation data, 476 counterfactual activations were classified as the targeted class. To test the robustness of the result against image corruption, we isolated the image components added by CAG (i.e., the difference between the counterfactual activation and the raw activation). Then, we perturbed the image components at different levels of percentile thresholds (α in Table 2. see methods) that were in turn added back to the raw activation. The effect of thresholding did not change the classification results when the bottom 20% of the image components were perturbed. The classification results were still above 25%, even when the bottom 60% of the image components were perturbed. The classification results were markedly degraded when the bottom 80% of the image components were perturbed. Thus, these results suggest that image modifications imposed by CAG were robust to perturbation in a large margin. To further assess the effectiveness of counterfactual explanation, we compared the classifier's response to counterfactual activations and control maps obtained by adding the original activation and the difference of average activations (“Control” in Table 2). Only four of the control maps were classified as the targeted class. Together, these results demonstrated that counterfactual explanations provided interpretable activation patterns that could not only explain the classifier's decisions but also robustly manipulate the classifier's decisions.

**Table 2:** Decision of DNN classifier on counterfactual activations obtained from correctly classified brain activations.

**Correct cases**	**Counterfactual activations**						**Control**
	**α = 0**	**α = 20**	**α = 40**	**α = 60**	**α = 80**	**α = 100**	
N_correct_ (%)	476 (99.6%)	470 (98.0%)	327 (68.4%)	125 (26.2%)	9 (1.9%)	0 (0.0%)	4 (0.8%)

*Counterfactual activations were obtained from images correctly classified by the DNN classifier (N = 478). Each image was transformed to one of a number of randomly chosen incorrect classes. All counterfactual activations were classified as the targeted class by the DNN classifier (middle column). As for the control analysis, the difference of the average maps for targeted vs. original classes was added to each image. None of the control images were classified as the targeted class (right column)*.

Note that the aim of the discussion here is not to infer cognitive tasks associated with the brain activation, a type of discussion considered as reverse inference (Poldrack, 2006). In this case, the cognitive tasks (i.e., classes) associated with the brain activations were entirely determined by the DNN classifier. The purpose of the discussion here is to interpret the counterfactual explanation in relation to existing knowledges about the brain activity. In the future, this type of discussion may be automated using applications such as Neurosynth (Yarkoni et al., 2011).

### Counterfactual Explanation of Misclassification by DNN Classifiers

**Figure 5:**
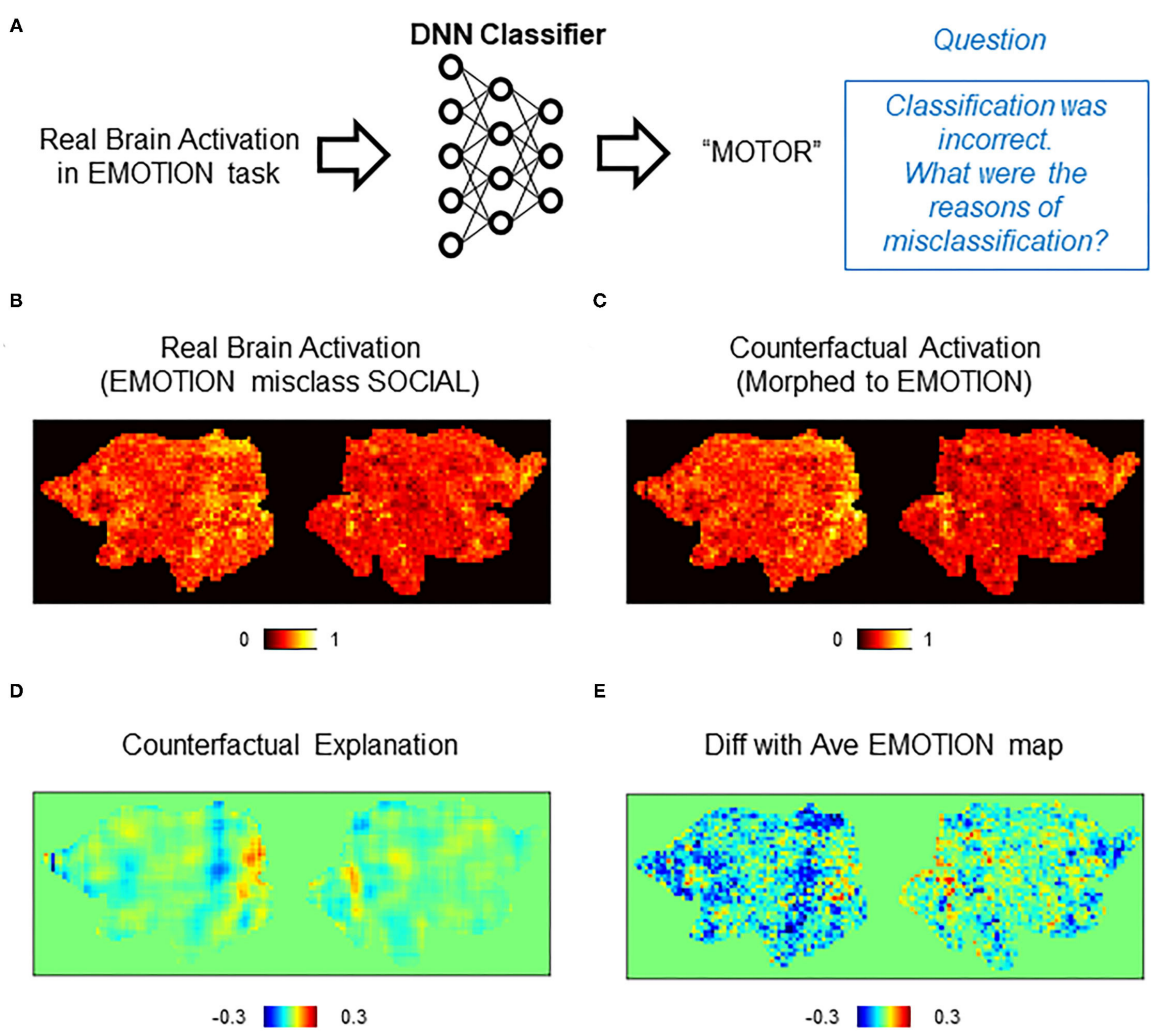
Counterfactual explanation of incorrect classification. **(A)** Schematics of the question asked in this analysis. In this example, the DNN classifier incorrectly assigned a “SOCIAL” label to a real brain activation in the EMOTION task. Here, we want to interrogate this incorrect decision. Specifically, we ask a question “why did the classifier (incorrectly) assign EMOTION instead of SOCIAL?” **(B–E)** Example of counterfactual explanation. **(B)** A single brain activation map for EMOTION that was incorrectly classified as SOCIAL by the DNN classifier. **(C)** A map of counterfactual activation obtained by transforming the map in **(A)** to SOCIAL. **(D)** Pixel-by-pixel subtraction of maps in **(B)** and **(A)** that serves as counterfactual explanation. This map explains why the map was incorrectly classified as SOCIAL but not EMOTION. **(E)** Simple difference between the average of real activations in the EMOTION and the single activation map for SOCIAL shown in **(A)**.

An important feature of counterfactual explanation is its ability to provide explanations to single cases of misclassification. We next demonstrated this in misclassifications by the DNN classifier (Figure 5A). For each case of misclassifications, the misclassified activation map was transformed toward the correct class by CAG. Then, the difference between the counterfactual activation and the real (misclassified) activation was calculated for the counterfactual explanation. In the first example, a brain activation in the EMOTION task was incorrectly classified as SOCIAL (Figure 5B). A counterfactual activation was obtained by transforming the real activation toward the correct class (EMOTION) (Figure 5C). Interestingly, the counterfactual explanation suggested that activations in the occipital regions were critically lacking for the DNN classifier to classify the original activation as EMOTION (Figure 5D). Because the occipital area is considered to process low-level visual information (Yamins et al., 2014), this occipital activation likely indicates bias in the dataset that used visual stimulus in the EMOTION task (Barch et al., 2013) rather than a brain activation related to emotional processing. Thus, counterfactual explanation revealed that this misclassification was likely due to the bias in the dataset, which was unintentionally learned by the DNN classifier.

As for a control analysis that can be compared with the counterfactual explanation, we calculated the difference between the misclassified (real) activation and the average activation of EMOTION (Figure 5E). Despite the similar global trend with the counterfactual explanation, the difference with the average showed a noisy pattern whose local peaks were difficult to find. Importantly, a peak in the occipital area was difficult to discern in the difference with the average. In the second example, we examined an activation in WM that was misclassified as GAMBLING (Supplementary Figure 5A). A counterfactual activation was obtained by transforming the real activation toward WM (Supplementary Figure 5B). As in the first example, the counterfactual explanation showed a pattern of brain activation with multiple identifiable peaks (Supplementary Figure 5C). In contrast, the difference with the average provided a noisier pattern whose local peaks were difficult to identify (Supplementary Figure 5D). These results demonstrated that counterfactual explanation can provide interpretable patterns of brain activations related to individual cases of misclassifications by the DNN classifier.

Next, we quantitatively assessed the counterfactual explanation of misclassifications. The DNN classifier assigned the correct classes to all the counterfactual activations that are equivalent to additions of the real (misclassified) activations and the counterfactual explanations (40 of 40 misclassified activations in the validation set). To assess the robustness of the results to image perturbation, we conducted the same analysis that we used for the correct classification. In the case of misclassification, the DNN classifier assigned the correct classes in 80% of cases, even when the bottom 80% of the image components modified by CAG were perturbed (Table 3). This result suggests that only a small modification to the misclassified activation was necessary to shift the classifier's decision to the correct class.

**Table 3:** Decision of the DNN classifier on counterfactual activations obtained from misclassified brain activations.

**Incorrect cases**	**Counterfactual activations**						**Control**
	**α = 0**	**α = 20**	**α = 40**	**α = 60**	**α = 80**	**α = 100**	
N_correct_ (%)	40 (100%)	40 (100%)	39 (97.5%)	38 (95.0%)	24 (60.0%)	0 (0.0%)	2 (0.5%)

*Counterfactual activations were obtained from images originally misclassified by the DNN classifier (N = 40). All of the counterfactual activations were correctly classified by the DNN classifier after transformation by CAG (middle column). As for the control analysis, the difference of the average maps for incorrect and correct classes was added to each misclassified image. None of the control images were classified as the targeted class (right column)*.

As for the control analysis, for each misclassified activation, we calculated the control activation that is the sum of the misclassified activation and the difference of averages of the true class and the incorrectly assigned class. In contrast to counterfactual activations, only two of the control activations were classified as the true classes (Table 3). These results suggest that counterfactual explanation, but not the addition of the difference of average activations, captured the image transformation needed to correct the decisions of the DNN classifier.

### Counterfactual Exaggeration Revealed Subtle Image Features Important for the Classifications by DNN

**Figure 6:**
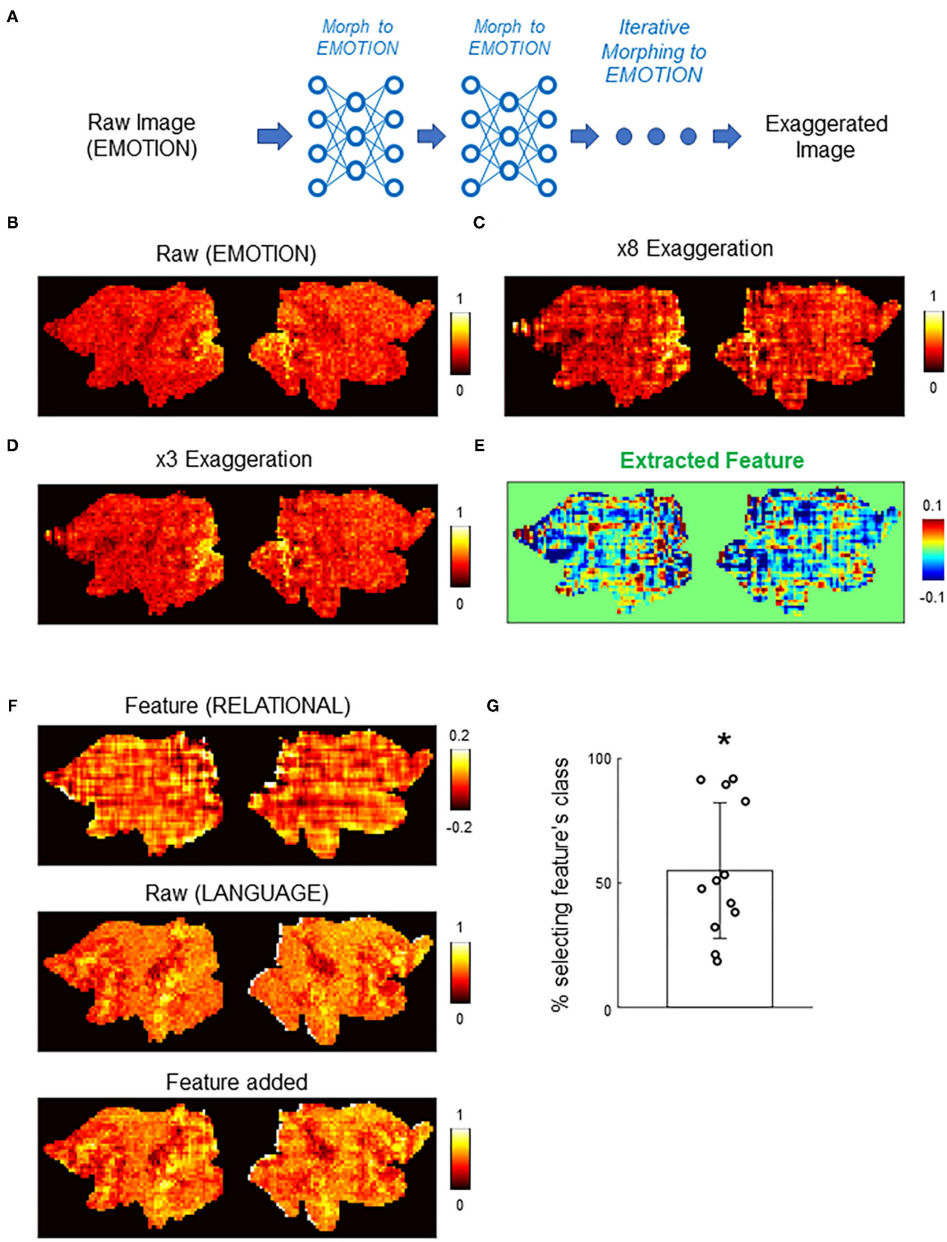
Counterfactual exaggeration of brain activation. **(A)** Schematic of counterfactual exaggeration. A brain activation (MOTOR task in this example) was iteratively transformed toward MOTOR by CAG. This iterative transformation accentuates (exaggerates) image features that biases the classifier decision toward MOTOR. **(B–D)** Example of counterfactual exaggeration. A brain activation in the EMOTION task **(B)** was iteratively transformed toward EMOTION eight times. Images after third **(C)** and eighth **(D)** transformations are shown. **(E)** Subtle image feature enhanced by counterfactual exaggeration was isolated by taking the difference of exaggerated images. In this example, differences between exaggerated images in **(C)** and **(D)** were calculated. The resulting difference image showed a texture-like pattern. **(F)** Example of texture-like feature extracted by counterfactual exaggeration (top). Bottom panel shows the texture-like patterns added to randomly chosen raw brain activations (middle). See also Supplementary Figure 6 for another example. **(G)** Decisions of the DNN classifier to brain activations with texture-like patterns added. Each dot represents one example texture (*N* = 12. Methods for details). Bar graph shows the mean and the standard deviation. The classifier was significantly biased toward the class of texture-like patterns (*, *p* < 0.001, Wilcoxon's sign rank test). Chance level was one of seven.

In addition to counterfactual explanations of correct and incorrect classifications, the deep image generator can perform “counterfactual exaggeration” to enhance and detect subtle image features exploited by DNNs (Singla et al., 2020). In counterfactual exaggeration, an image is iteratively transformed by the generator toward one class. This iterative image transformation enhances subtle image features exploited by DNNs. In a previous work, exaggerated images were used to discover a novel symptom of diabetic macular edema (Narayanaswamy et al., 2020). Inspired by these previous works, we next used CAG in counterfactual exaggeration to detect subtle features of brain activations exploited by the DNN classifier (Figure 6A). Interestingly, in some cases, iterative application of CAG revealed a texture-like feature in the image (Figures 6B–D). Such a texture-like feature was difficult to discern in the original activation (Figure 6B) but became evident as the counterfactual exaggeration was repeatedly applied (Figures 6C,D). The texture-like feature could be extracted by taking the difference in counterfactual activations with different numbers of iterations (Figure 6E).

Although the texture-like pattern did not appear in the same way as a real brain activation, it could nevertheless influence the classifier decisions. In fact, it has been suggested that DNNs are biased toward using textures for image classification (Geirhos et al., 2018). To quantitatively examine this point, we added the extracted features to randomly chosen real activations and then examined the resulting activations by the DNN classifier. Figure 6F and Supplementary Figure 6 show examples of the extracted features and the real activations before and after the addition of the features. Note that differences between the appearance of activations before and after the addition of the features were subtle because the amplitudes of the extracted features were relatively small. Nevertheless, the addition of the extracted features caused the DNN classifier to (mis-)assign the activations the classes to which the exaggerations were targeted (Figure 6G). Misclassification to the targeted class occurred in 55.0 ± 26.1% of cases (mean ± standard deviation; *N*=12 extracted features; *p* < 0.001, sign rank test; see Methods for details). These results suggest that counterfactual exaggeration assisted by CAG was able to enhance and discover subtle image features that are exploited by the DNN classifier. The texture-like features likely represent image features relevant to adversarial vulnerability of the DNN classifier (Geirhos et al., 2018). Being able to detect and protect against such attacks is critical for future reliable applications of DNN-based brain decoders.

## Discussion

In this study, we provided a proof-of-principle of application of counterfactual explanations from a generative model to understand the decisions of a DNN trained to decode task information from brain activation data. In the field of computer vision, such explanation is often conducted with saliency maps that highlight regions in the input that were important for the decisions of the DNN (Selvaraju et al., 2020). A recent neuroimaging study also used saliency maps to interpret decisions made by DNNs (Tsumura et al., 2021). A limitation of this approach is that the regions highlighted in saliency maps are not necessarily causally related to the decisions of the DNN (Eitel and Ritter, 2019). Hence, user of saliency maps needs to perform an additional interpretation of why the highlighted areas are important for the DNN's decisions (Mertes et al., 2020). In contrast to saliency maps, counterfactual explanation explains why the actual decision was made instead of another one. By creating a slightly modified version of the input that leads another decision by the DNN, counterfactual explanation provides a different kind of explanatory information which helps to interpret saliency maps. Thus, future neuroimaging studies and also brain machine interface studies using DNNs [e.g., Willett et al. (2021)] can combine counterfactual explanation with saliency maps to better interpret how the patterns of brain activations are causally related to DNN decisions.

There are several limitations in this study. The training and testing of the DNN classifier and CAG were performed using only the HCP dataset. As more and more neuroimaging datasets become available to the public, researchers are starting to develop DNN classifiers trained on multiple datasets. Though it is beyond the scope of this study, explaining the DNN classifiers trained on multiple datasets would be an important future research topic. Another limitation is that this study used spatial downsampling to enable efficient learning by CAG. This was partly due to limitations in both the computational power and the dataset size. The limitation in the dataset size may be alleviated using techniques for data augmentation (Shorten and Khoshgoftaar, 2019).

It should also be emphasized that the aim of CAG is not to improve the accuracy of the DNN classifier but to provide visual explanations for the classifier's decisions. Because CAG can simultaneously take into account information from the entire brain, counterfactual explanation is different from conventional analyses of local activation patterns such as GLM and search light-based multivariate pattern analyses (Kriegeskorte et al., 2006; Jimura and Poldrack, 2012; Chikazoe et al., 2014). This characteristic of CAG is most pronounced in counterfactual exaggerations, where it discovered global texture-like patterns that could effectively bias the classifier's decisions. At present, these patterns are unlikely to reflect biologically important activity patterns. Further development of CAG and related techniques would enable the discovery of global activity patterns with biological significance beyond conventional analyses.

### Conclusions

In this study, we developed CAG, a generative neural network for counterfactual brain activation that can be used to explain individual decision behaviors of DNN-based classifiers. A single CAG could handle multiple classes at the same time and learn mapping between all the pairs of classes. CAG could provide visually intuitive counterfactual explanations for a classifier's correct and incorrect decisions. These counterfactual explanations were quantitatively more effective in explaining the classifier's decision than the controls and were robust against image perturbations. Finally, beyond explaining the decision behaviors, CAG could extract subtle image features in the brain activation that were invisible to the eyes but that were exploited by the DNN classifiers. Together, these results suggest that counterfactual explanation with CAG provides a novel approach to examine and extend current neuroimaging studies using DNNs.

## Data Availability Statement

Publicly available datasets were analyzed in this study. This data can be found here: S1200 release of the Human Connectome Project (http://www.humanconnectomeproject.org).

## Author Contributions

TM and MT conceived the study conducted analysis. KJ conducted analysis and provided reagents. TM wrote the manuscript with inputs from all authors. All authors discussed the results.

## Funding

This study was supported by JSPS Kakenhi (20H05052 and 21H0516513 to TM, 19K20390 to TP, 19H04914 and 20K07727 to KJ, 21H02806 and 21H05060 to JC), a grant from Japan Agency for Medical Research and Development (AMED) to JC (Grant Number JP19dm0207086), a grant from Brain/MINDS Beyond (AMED) to TM and MT (Grant Number JP20dm0307031), a grant from JST-PRESTO to TM, a grant from Narishige Neuroscience Research Foundation to TM.

## Conflict of Interest

JC is Employed by Araya Inc. The remaining authors declare that the research was conducted in the absence of any commercial or financial relationships that could be construed as a potential conflict of interest.

## Publisher's Note

All claims expressed in this article are solely those of the authors and do not necessarily represent those of their affiliated organizations, or those of the publisher, the editors and the reviewers. Any product that may be evaluated in this article, or claim that may be made by its manufacturer, is not guaranteed or endorsed by the publisher.
