# Supplementary material for: Counterfactual Explanation of Brain Activity Classifiers Using Image-To-Image Transfer by Generative Adversarial Network
Source: Front Neuroinform. 2022 Mar 16;15:802938. doi: 10.3389/fninf.2021.802938 (PMC8966478; doi:10.3389/fninf.2021.802938)
Supplement: Supplementary file 1 [file Data_Sheet_1.docx]

Supplementary Material

##
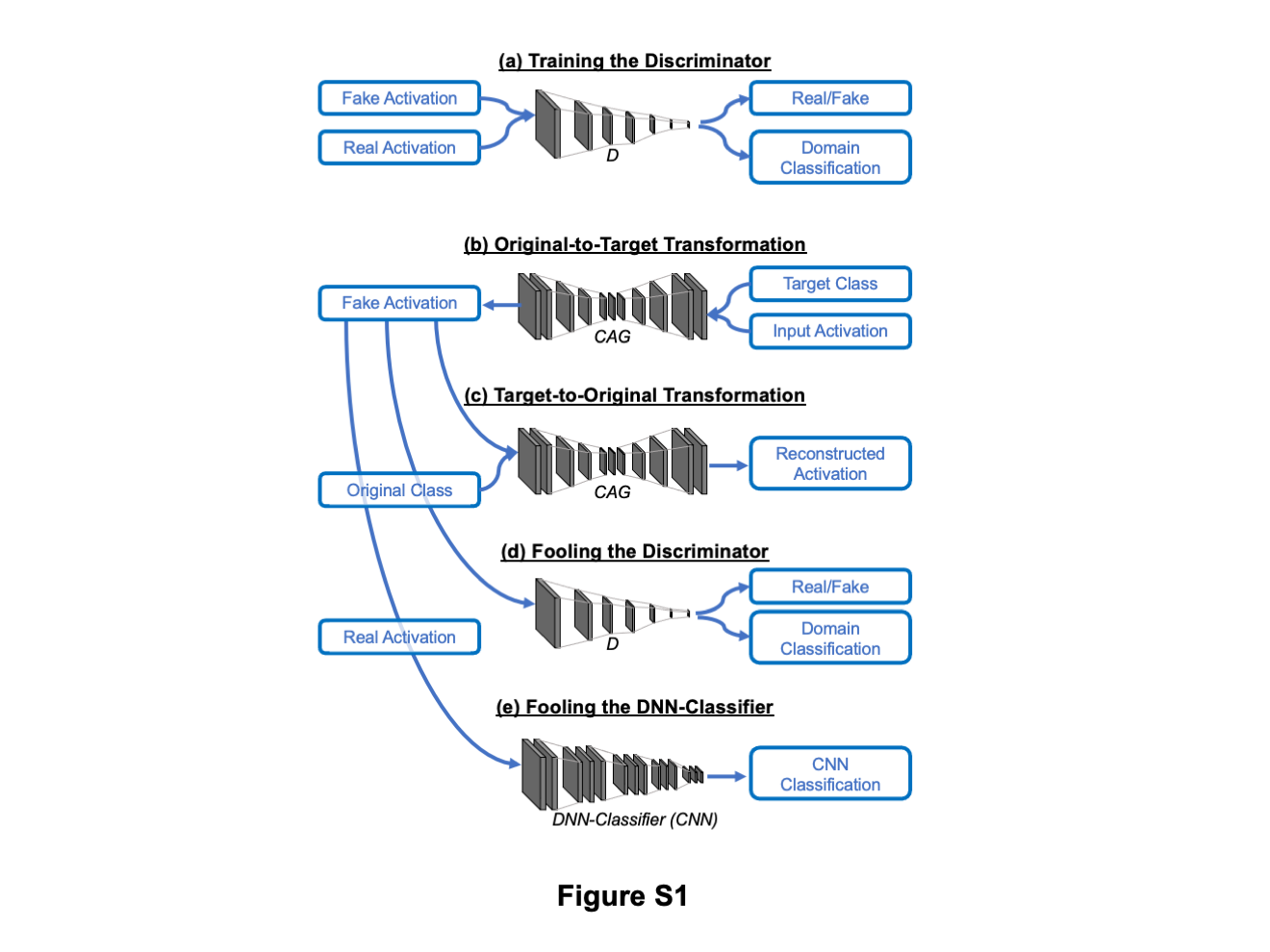
Supplementary Figures

## Figure S1. Overall architecture of Discriminator/CAG training. We adapted the overall design of the StarGAN(Choi *et al.*, 2018). The architecture includes two trainable modules, Discriminator (D) and CAG. Note that DNN-classifier is not trainable. (a) D learns to discriminate between real and fake activations and to classify the real activation to its corresponding domain. (b) CAG takes a real activation and a target domain label (*i.e.* target class) to generate a fake activation. (c) CAG tries to reconstruct the original activation from the fake activation and the domain label of the original activation. (d) CAG tries to generate a fake activation that is indistinguishable from real activations and classifiable as target domain by D. We also added a process “Fooling the DNN-Classifier” (e) to force CAG to generate a fake activation that is classifiable as target domain by the DNN-classifier.

**
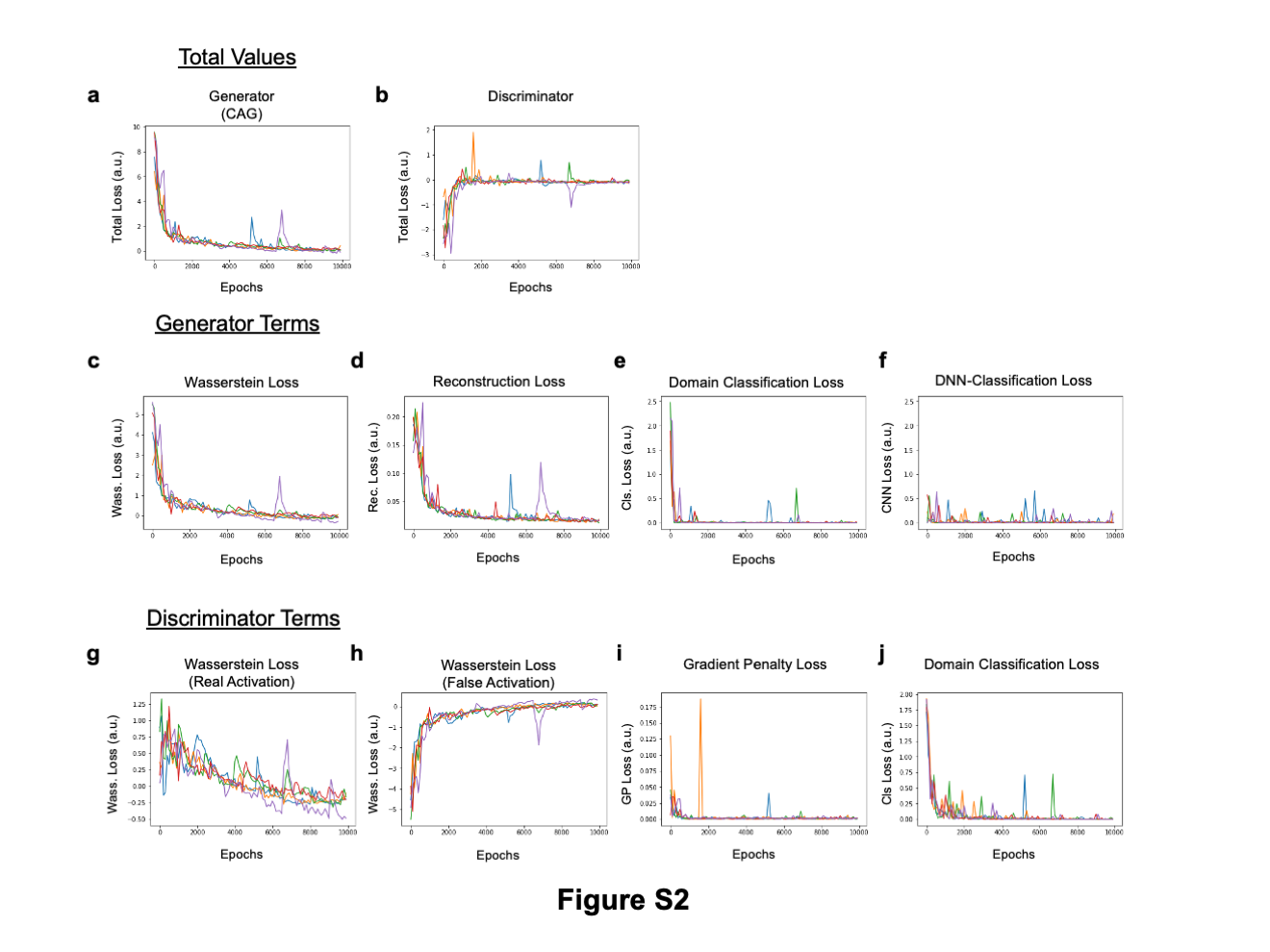
**

## Figure S2 Time courses of key terms related to the Discriminator/CAG training. a) Total Generator Loss for CAG (same as Fig.3b). b) Total Discriminator Loss for Discriminator. c-f) Decomposed Generator Loss. g-j) Decomposed Discriminator Loss. Five independent replicates are shown in different colors.


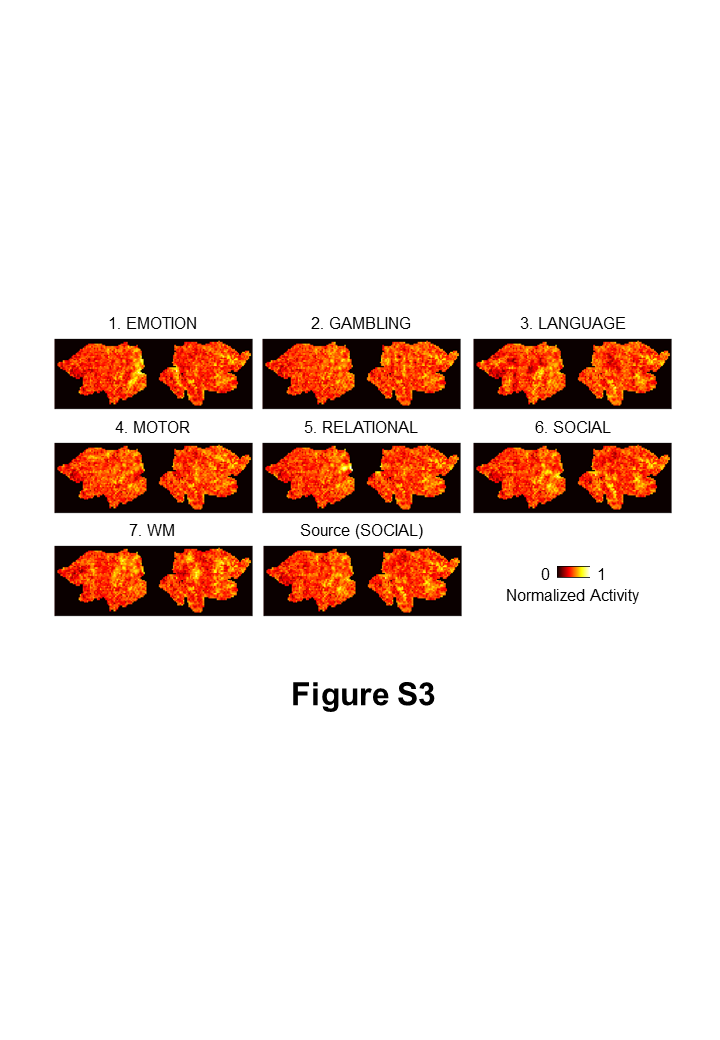
**Figure S3 Representative counterfactual activations generated by CAG (related to Fig. 3c).** All counterfactual activations were generated from the source activation.


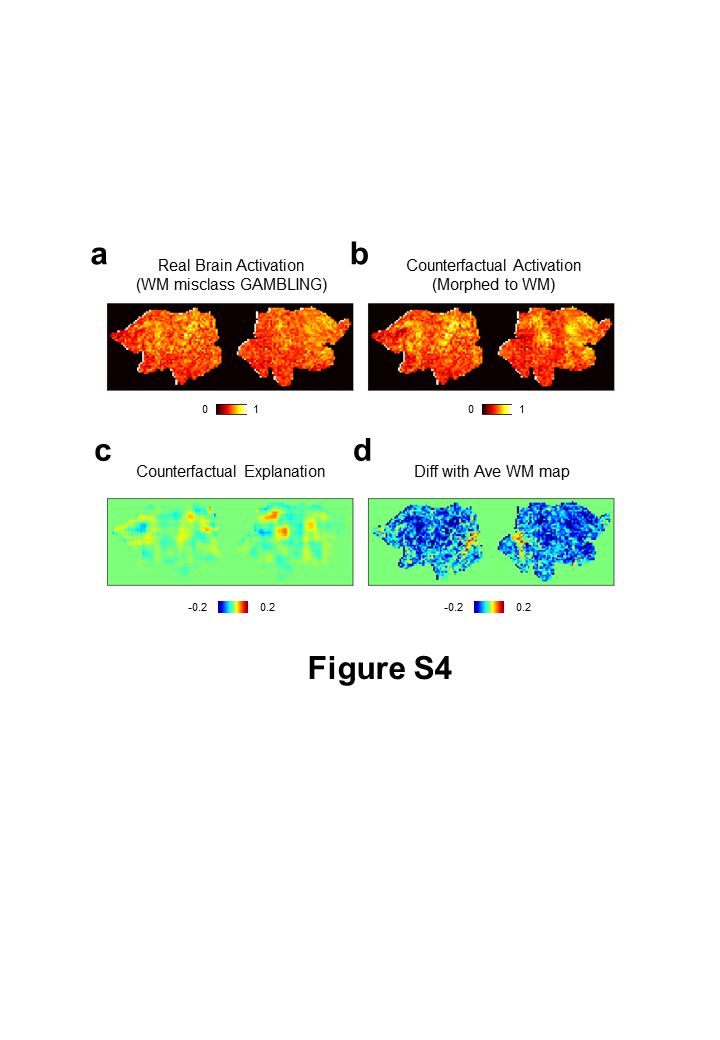


**Figure S4. Additional example of counterfactual explanation of correct classification.** Same convention as in Fig.4(b)-(e). In this case, we asked why brain activations were (correctly) classified as WM but not LANGUAGE.


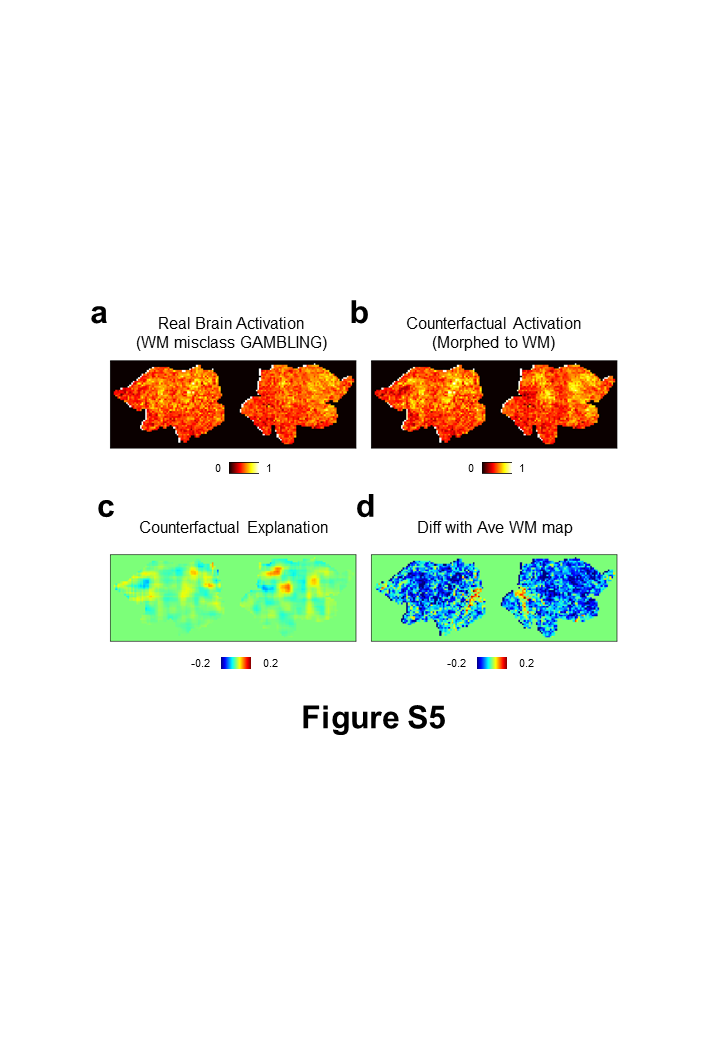


**Figure S5. Additional example of counterfactual explanation of incorrect classification.** Same convention as in Fig.5(b)-(e). In this case, we asked why brain activations were (incorrectly) classified as GAMBLING but not WM.


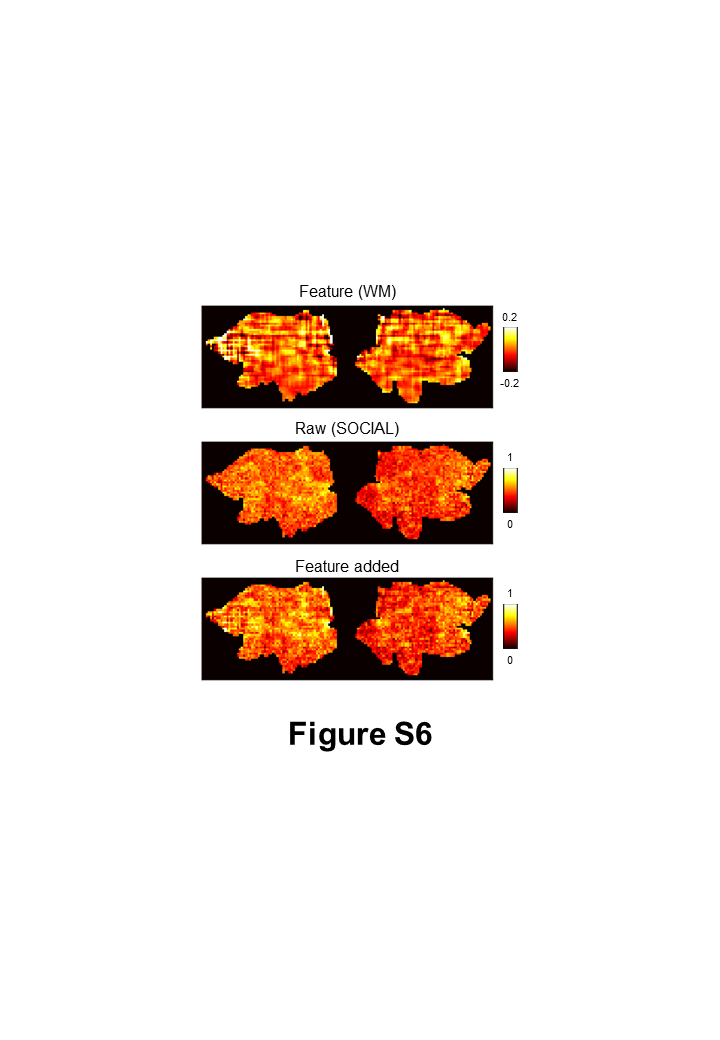


**Figure S6. Another** example of texture-like feature extracted by counterfactual exaggeration (related to Fig. 6f). Top, texture-like feature extracted by counterfactual exaggeration. Middle, randomly chosen raw brain activations. Bottom, texture-like patterns added to the raw brain activation shown in the middle.

**Supplementary Table**

|  | | Output Class | | | | | | |
| --- | --- | --- | --- | --- | --- | --- | --- | --- |
|  |  | EMOTION | GAMBLING | LANGUAGE | MOTOR | RELATIONAL | SOCIAL | WM |
| Input Class | EMOTION | 0.986 | 0 | 0 | 0 | 0 | 0.014 | 0 |
|  | GAMBLING | 0 | 0.877 | 0 | 0 | 0.068 | 0.055 | 0 |
|  | LANGUAGE | 0 | 0 | 0.986 | 0 | 0 | 0.014 | 0 |
|  | MOTOR | 0 | 0 | 0 | 1 | 0 | 0 | 0 |
|  | RELATIONAL | 0 | 0.095 | 0 | 0.027 | 0.851 | 0.014 | 0.014 |
|  | SOCIAL | 0.014 | 0.027 | 0 | 0 | 0.027 | 0.932 | 0 |
|  | WM | 0 | 0.107 | 0 | 0.027 | 0.04 | 0 | 0.827 |

**Table S1. Profile of the DNN classifier (Confusion Matrix)** Same as Fig.2d but exact numbers for proportion correct are shown)
